# Selection of Honey Bee (*Apis mellifera*) Genotypes for Three Generations of Low and High Population Growth of the Mite *Varroa destructor*

**DOI:** 10.3390/ani14233537

**Published:** 2024-12-07

**Authors:** Alvaro De la Mora, Paul H. Goodwin, Berna Emsen, Paul G. Kelly, Tatiana Petukhova, Ernesto Guzman-Novoa

**Affiliations:** 1 School of Environmental Sciences, University of Guelph, 50 Stone Road East, Guelph, ON N1G 2W1, Canada; delamora@uoguelph.ca (A.D.l.M.); pgoodwin@uoguelph.ca (P.H.G.); bernaemsen@gmail.com (B.E.); pgkelly@uoguelph.ca (P.G.K.); 2 Department of Population Medicine, University of Guelph, 50 Stone Road East, Guelph, ON N1G 2W1, Canada; tpetukho@uoguelph.ca

**Keywords:** *Apis mellifera*, *Varroa destructor*, selective breeding, mite resistance, colony collapse disorder, deformed wing virus

## Abstract

Honey bee (*Apis mellifera*) population declines have been associated with the parasitic mite, *Varroa destructor*, which is currently primarily controlled by the use of acaricides. An alternative is to breed for resistance to *Varroa*, which was conducted in this study by bidirectional selection for mite fall to obtain colonies with low (resistant) or high (susceptible) *Varroa* population growth (LVG and HVG, respectively). Selection for three generations resulted in approx. 90% lower *Varroa* population growth in LVG than in HVG colonies. In addition, late summer *Varroa* infestation rates of brood and adults were both significantly lower in LVG colonies (*p* < 0.01), which was also significantly associated with lower Deformed Wing Virus (DWV) infection levels (*p* < 0.01). Survival of *Varroa*-parasitized bees was almost 50% higher for LVG bees compared to HVG bees (*p* < 0.01). Also, colony winter survivorship was significantly higher for LVG colonies than for HVG colonies (*p* < 0.05). However, the higher colony populations observed for LVG colonies were not significantly different from those of HVG colonies. Overall, individual and colony health was improved by selecting colonies for LVG, demonstrating its effectiveness as a means of breeding for controlling *Varroa* populations in honey bee colonies.

## Simple Summary

One of the main culprits of honey bee colony losses is the parasitic mite, *Varroa destructor*, which is primarily controlled with acaricides, which lose efficacy due to resistance and can contaminate honey. An alternative is to breed bees that are resistant to *Varroa*, which was conducted in this study by bidirectional selection for mite fall to obtain colonies with low (resistant) or high (susceptible) *Varroa* population growth (LVG and HVG, respectively). Selection for three generations resulted in greatly reduced *Varroa* population growth in LVG compared to HVG colonies. In addition, *Varroa* infestation rates of bees were lower in LVG colonies, and they had lower Deformed Wing Virus (DWV) infection levels. Survival of *Varroa*-parasitized bees was higher for LVG bees compared to HVG bees, which may help explain why colony winter survivorship was higher for LVG colonies than for HVG colonies. Selecting colonies of bees for LVG resulted in better individual and colony bee health, demonstrating its effectiveness as a means of breeding for controlling *Varroa* mites.

## 1. Introduction

The mite *Varroa destructor* is recognized as the most harmful parasite of the western honey bee (*Apis mellifera* L.) and is associated with high colony losses worldwide, mainly in the northern hemisphere [1]. *Varroa* primarily feeds upon the fat body tissue and hemolymph of honey bees [2,3], and reduces honey bee weight, immune responses, lifespan, and honey production [4,5,6,7]. A major factor in the detrimental effects of *Varroa* is its saliva, which contains viruses, including Deformed Wing Virus (DWV). It also contains *Varroa* toxic protein, which can increase DWV levels in the bee [1]. DWV can replicate within the mite and be transmitted by the parasite to honey bees [8]. DWV infection sometimes causes deformed bodies and wings in broods and is frequently associated with shortened lifespans in adults [9,10,11,12]. Hence, both *Varroa* and DWV are linked to honey bee mortality and colony losses. Furthermore, the winter conditions of northern climates, like those of Canada, magnify the impact of *Varroa* and DWV [12,13].

Most beekeepers control colony *Varroa* infestations with synthetic acaricides, but the parasite can develop resistance to them, which compromises their efficacy [14]. Accordingly, it is necessary to have alternate control strategies, one of which is breeding for *Varroa* resistance [15,16]. One mechanism that has been associated with resistance to *Varroa* is grooming behavior, where bees bite and dislodge mites from their bodies, and this has been used for selecting honey bee stocks, such as the ‘mite-biter’ genotype [17,18]. Another mechanism is hygienic behavior, by which bees identify and remove not only diseased or dead brood from their cells, but also *Varroa*-infested brood, and it has been used for the selection of honey bee stocks, such as the *Varroa*-sensitive hygiene genotype [19,20]. A third option for increasing *Varroa* resistance is selecting bee genotypes for lower mite infestation levels, which can be determined by mite fall in a colony to measure mite infestation levels over time (i.e., lower *Varroa* population growth), such as for the ‘Arbeitsgemeinschaft Toleranzzucht’ (AGT), Primorski Russian, and low *Varroa* growth (LVG) genotypes [21,22,23,24].

In the selection program to produce LVG bees, De la Mora et al. [24] showed that two generations of selection for low rates of *Varroa* population growth resulted in colonies with significantly lower mite population growth, mite infestation rates in adult bees and brood, winter colony mortality, and DWV levels, compared to colonies selected for high rates of *Varroa* population growth (HVG). However, the authors did not assess colony bee populations and worker bee survivorship when parasitized with mites. Also, the bees in that study were not inoculated with *Varroa* to ensure parasitism, and thus it was not possible to determine if the resistance was due to less *Varroa* in the colonies and thus avoidance of parasitism, versus an antiviral mechanism within parasitized bees. The current study reports results from a third generation of selection for LVG and HVG assessing the variables of the previous study [24], as well as colony populations and the impact of *Varroa* parasitism on honey bee survivorship. As a result, this study provides a more comprehensive view of the effects of selecting for LVG combined with an additional generation of selection.

## 2. Materials and Methods

### 2.1. Selective Breeding Procedures for LVG and HVG Genotypes

The breeding program was conducted at the Honey Bee Research Centre (HBRC), University of Guelph, Guelph, ON, Canada (43.5448° N, 80.2482° W). The selective breeding procedure was conducted following previous studies [24,25]. Briefly, mite populations in colonies were determined by counting the number of *Varroa* mites fallen on sticky boards over three days, which was then divided by three to determine the average per day. Mite population growth was determined by comparing the number of fallen mites in early spring (May), and 15 weeks later in late summer (August). Three colonies with the lowest *Varroa* population growth (LVG) and three with the highest *Varroa* population growth (HVG) were selected for each generation as per De la Mora et al. [24]. These colonies were treated against *Varroa* with amitraz strips (Apivar^®^, Véto-Pharma, Palaiseau, France) during early fall to ensure their survival during the winter. The following spring, more than 100 queens were raised for each genotype, grafting larvae from the selected colonies to create each generation. The queens reared were allowed to open mate at a common mating yard, which was isolated at least 5 km from other apiaries and surrounded by drone-producing colonies of the Buckfast strain. To produce each generation, at least 100 colonies over 10 apiaries were dequeened, and then each was equally divided into two halves with one half receiving an LVG queen in a brood chamber and the other half receiving a HVG queen in another brood chamber. This practice guaranteed similar starting mite levels as well as colony populations and apiary conditions for both genotypes. The colonies derived from the introduced queens were managed the same way throughout the season and tested for *Varroa* population growth as previously described.

### 2.2. Varroa destructor Infestation Rates in Adult Bees and Brood

Twenty randomly selected colonies per genotype of the third generation were assessed for *Varroa* infestation rates in adult bees and brood based on the number of attached mites per 100 bees and the number of brood cells infested with mites per 100 cells, respectively. These assessments were conducted at the end of the summer. For adults, approximately 300 bees were collected from the brood nest of each colony, placed in 100 mL of 70% ethanol, and processed as per De Jong et al. [26] to determine the number of mites per 100 bees. For brood, approximately 200 worker brood cells containing pupae with pink-purple eyes were uncapped, and the percentage of mite-infested brood per 100 cells was calculated [27].

### 2.3. DWV Infection Levels

At least 15 adult bees were collected in late summer from the brood nest of three randomly selected colonies of each genotype from the third generation of selection. The bees were immediately frozen at −80 °C. Quantitative real-time PCR (qRT-PCR) of DWV was performed as per Morfin et al. [28] with modifications for RNA extraction. Briefly, 15 frozen bees from each sample were macerated with 5 mL of One Step RNA Reagent (BioBasic, Markham, ON, Canada) following instructions from the manufacturer. The macerate was transferred to a new 1.5 mL centrifuge tube and incubated for 5 min at 20–22 °C. Then, 300 µL of chloroform was added, and the tube was vortexed at 7000 rpm for 15 s. After incubation at 20–22 °C for 2–3 min, the tube was centrifuged (Symphony 417R, VWR, Mississauga, ON, Canada) at 12,000× *g* for 15 min at 4 °C. The aqueous phase was transferred to a new 1.5 mL microcentrifuge tube, and 500 µL of 99% isopropanol was added. The tube was incubated at 20–22 °C for 10 min and then centrifuged at 12,000× *g* for 10 min at 4 °C. The RNA pellet was washed with 1 mL of 70% ethanol, mixed by inversion, and centrifuged at 7500× *g* for 5 min at 4 °C. This was repeated two additional times. After drying, the RNA pellet was dissolved in 30 µL of Invitrogen UltraPure H_2_O (Fisher Scientific, Waltham, MA, USA), and the RNA quality was assessed using a NanoDrop Lite (Thermo Fisher, Waltham, MA, USA). RNA samples were stored at −80 °C.

cDNA was prepared by using 2 mg of RNA per sample and a Fermentas Revert Aid H Minus First Strand cDNA Synthesis Kit (Fisher Scientific, Burlington, ON, Canada), following the manufacturer’s instructions. cDNA was amplified with a QuantStudio3 thermocycler (Real-Time PCR Systems, Fisher, Pittsburgh, PA, USA). The forward primer for DWV type A was 5′ GCGCTTAGTGGAGGAAATGAA 3′ and the reverse primer was 5′ GCACCTACGCGATGTAAATCTG 3′ [29]. Each qPCR reaction consisted of 20 µL containing 2 µL cDNA, 0.4 µL forward and reverse primers (200 nM), 10 µL PowerUp^TM^ Sybergreen (2×) (Applied Biosystems, Foster City, CA, USA), and 7.2 µL nuclease-free H_2_O. The negative control was 2 µL of nuclease-free H_2_O instead of cDNA. The positive control was a DWV-positive honey bee cDNA sample previously identified. PCR conditions consisted of 1 cycle at 48 °C for 15 min and 95 °C for 10 min, followed by 40 cycles at 95 °C for 15 s and 60 °C for 60 s, and then 1 cycle at 68 °C for 7 min. Calibration curves to convert Ct values to DWV genome copies (gc) were conducted using 300 bp gBlocks (Integrated DNA Technologies, Coralville, IA, USA) that included the sequences of the forward primer, amplicon, and reverse primer. The lyophilized gBlocks were diluted in dsH_2_O to make 10-fold serial dilutions from 10^9^ to 10^2^ copies. Using a plot of Ct values versus DWV copy number (log10), a linear equation was used to calculate the DWV genome copy number [30]. Three technical repetitions were conducted for each sample. Randomly selected amplicons of presumed DWV were sequenced at the University of Guelph Laboratory Services to confirm identity.

### 2.4. Colony Populations and Winter Colony Mortality

The twenty randomly selected colonies per genotype that were sampled to assess mite levels were also used to evaluate adult bee populations in late summer. Briefly, a visual estimate of the area covered by bees in each frame (from 0 to 1) was obtained and then multiplied by 880, which corresponds to the total comb area (cm^2^) of a deep Langstroth frame. The result was then multiplied by the number of bees per cm^2^ (1.38) [31]. The survivorship of colonies of both genotypes that were overwintered was verified visually in late April to determine the percentage of colonies that were alive or dead.

### 2.5. Individual Bee Survivorship to Mite Parasitism

Female mites of *Varroa* were collected from highly infested colonies that were unrelated to the experimental bees by detaching the parasites from adult bees that were anesthetized with CO_2_ as per Dietemann et al. [27]. Collected mites were placed with a fine paintbrush in a plastic Petri dish lined with a moist paper towel until needed.

Two frames covered with workers were retrieved from the center of each of three third-generation colonies per genotype and then shaken into a 5 L container, from which nurse bees were collected. Ten bees from each colony were introduced into each of five wooden Benton cages (75 × 25 × 16 mm) for a total of 50 bees per treatment. The bees in each cage were subjected to one of four treatments: (1) LVG bees without *Varroa*; (2) LVG bees with *Varroa*; (3) HVG bees without *Varroa*; (4) HVG bees with *Varroa*. The experiment was replicated three times using a different source colony per replicate. For *Varroa* exposure, bees were artificially parasitized with one mite per individual by placing a mite with a fine paintbrush onto each bee, and the cages were maintained at 32–35 °C and 60% RH for eight days [7]. The bees were fed queen candy (glucose mixed with powdered sugar) and watered twice a day by placing clean sponges with dsH_2_O on the cage screen. Honey bee mortality was recorded, and dead bees were removed from the cages daily.

### 2.6. Statistical Analyses

Data were analyzed for normality with the Shapiro–Wilk test. When not normally distributed, the data were transformed. Data on *Varroa* population fold change and DWV copies were log-transformed, data on mite infestation rates on adult bees and brood were arcsine-square root transformed, and data on bee populations were not transformed. The data for mite population fold change in three generations were subjected to analysis of variance and Fisher LSD tests to separate means. Means for the other variables were analyzed with Student’s *t*-tests to compare genotypes. Winter colony mortality rate was analyzed with a 2-sample test for equality of proportions with continuity correction to test the alternative hypothesis that the proportion of dying colonies of the HVG genotype is greater than the proportion of dying colonies of the LVG genotype, based on results of a previous study with these same genotypes [24]. Additionally, the lifespan of individual bees of both genotypes with and without *Varroa* was subjected to survival analysis using the Kaplan–Meier method, along with the log-rank/Mantel-Cox post hoc test to compare the survival curves. Statistical analyses were performed with the R 4.1.1.software (R Development Core Team, Auckland, New Zealand).

## 3. Results

### 3.1. Varroa destructor Population Growth

**Figure 1:**
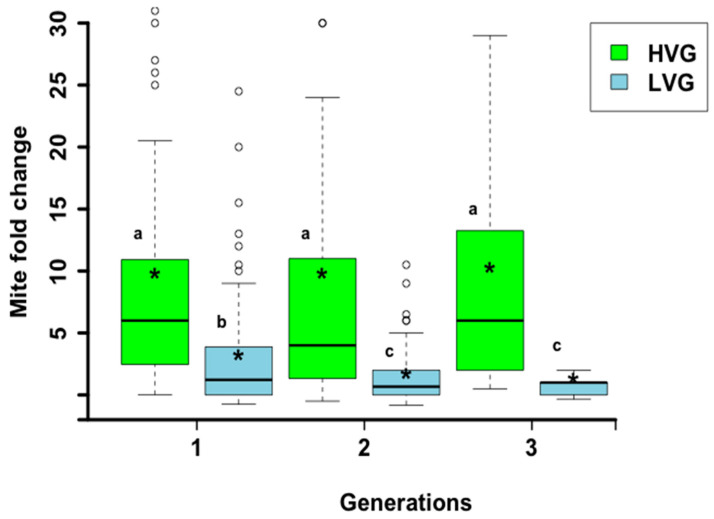
*Varroa destructor* population growth (mean fold change) during 15 weeks in honey bee colonies selected for three generations for high and low *Varroa* population growth (HVG and LVG, respectively). Boxes indicate the upper and lower quartiles, median is indicated by the solid line within the quartile box, means are indicated by the asterisk, and open circles indicate outliers. Different literals indicate significant differences based on analysis of variance and Fisher-protected LSD tests of log-transformed data. Untransformed values are shown.

Colonies of the HVG and LVG genotypes differed significantly from each generation of selection in *Varroa* population growth (F_1,690_ = 74.5, *p* < 0.0001; Figure 1) and there was a significant interaction between genotype and generation (F_3,688_ = 16.3, *p* < 0.0001). There were no significant differences between generations for HVG colonies, but the second and third generations of LVG bees had a significantly lower mite fold change than the first generation. The variation was greater for each generation of HVG than LVG colonies, and the degree of variation remained similar for each generation of HVG colonies, whereas it declined with each generation of LVG colonies, with less skewing in the direction of higher mite fold change. By the third generation, *Varroa* population growth for the LVG colonies was 89.7% lower than that of the HVG colonies, compared to 66.7% and 82.3% in the first and second generations, respectively.

### 3.2. Varroa destructor Infestation Rates in Adult Bees and Brood

**Figure 2:**
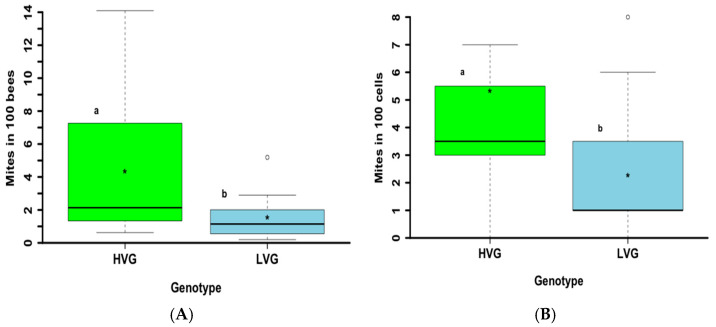
*Varroa destructor* late summer infestation rate in adult workers (**A**) and brood (**B**) from honey bee colonies of the third generation of selection for high and low *Varroa* growth (HVG and LVG, respectively). Boxes indicate the upper and lower quartiles, median is indicated by the solid line within the quartile box, means are indicated by the asterisk, and open circles indicate outliers. Genotypes differed significantly based on Student’s *t*-tests of arcsine-square root transformed data. Differences are indicated with different literals (a, b). Untransformed values are shown.

LVG colonies of the third generation of selection had a significantly lower *Varroa* infestation of adult bees than HVG colonies (*t*_38_ = 3.5, *p* < 0.001) (Figure 2A). Similar results were obtained for mite infestation of brood (*t*_38_ = 2.7, *p* < 0.01) (Figure 2B). Mite infestation was on average about three times higher in adult bees and two times higher in the brood of HVG colonies compared to LVG colonies with a much higher level of variability for adults but similar levels for brood between HVG and LVG colonies.

### 3.3. DWV Infection Levels

**Figure 3:**
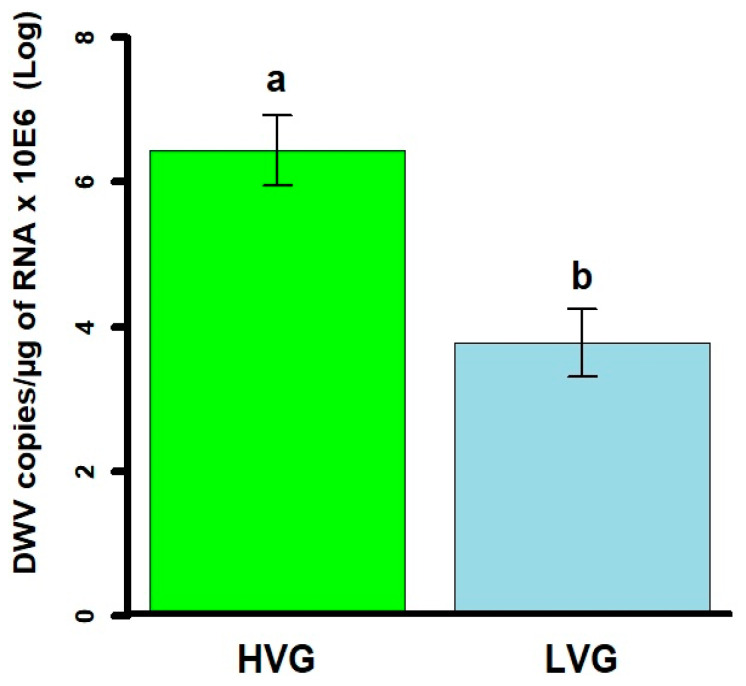
DWV infection levels (mean DWV copies/µg of RNA × 10^6^ ± SEM) in adults from honey bee colonies of the third generation of selection for high and low *Varroa* growth (HVG and LVG, respectively). Genotypes differed significantly based on Student’s *t*-tests of log-transformed data. Differences are indicated with different literals (a, b).

Adult bees from LVG colonies had Log 3.9 ± 0.4 DWV copies/µg of RNA × 10^6^ which was significantly lower than Log 6.5 ± 0.5 DWV copies/µg of RNA × 10^6^ of bees from HVG colonies (*t* = 4.0, *p* < 0.001; Figure 3).

### 3.4. Colony Populations and Winter Colony Mortality

**Figure 4:**
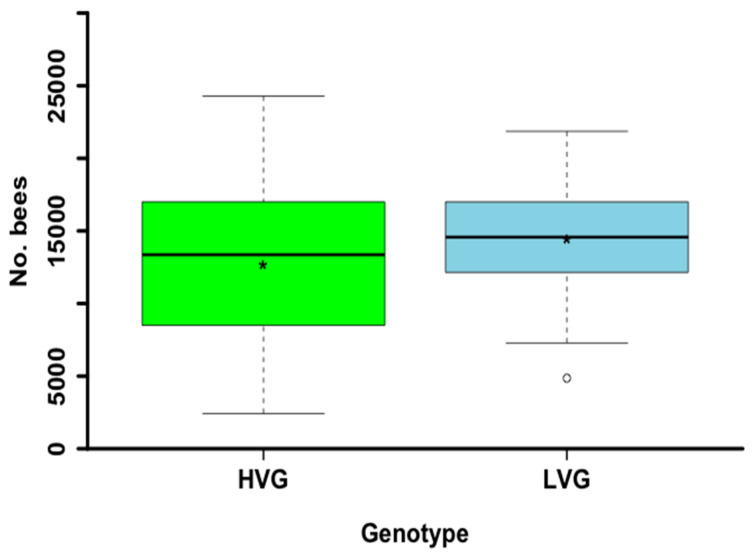
Summer adult bee populations of honey bee colonies of the third generation of selection for high and low *Varroa* mite population growth (HVG and LVG, respectively). Boxes indicate the upper and lower quartiles, median is indicated by the solid line within the quartile box, means are indicated by the asterisk, and open circles indicate outliers.

There were no significant differences for adult bee colony populations between the two genotypes at the end of the summer (*t*_38_ = −1.1, *p* = 0.276; Figure 4). However, based on the statistical evidence for the proportion of surviving colonies the following spring, the mortality of LVG colonies (*n* = 71) was 5.6%, which was significantly lower than the 15.4% mortality for HVG colonies (*n* = 78) (χ^2^ = 2.74, df = 1, *p* < 0.05).

### 3.5. Survivorship of Parasitized and Non-Parasitized Bees

**Figure 5:**
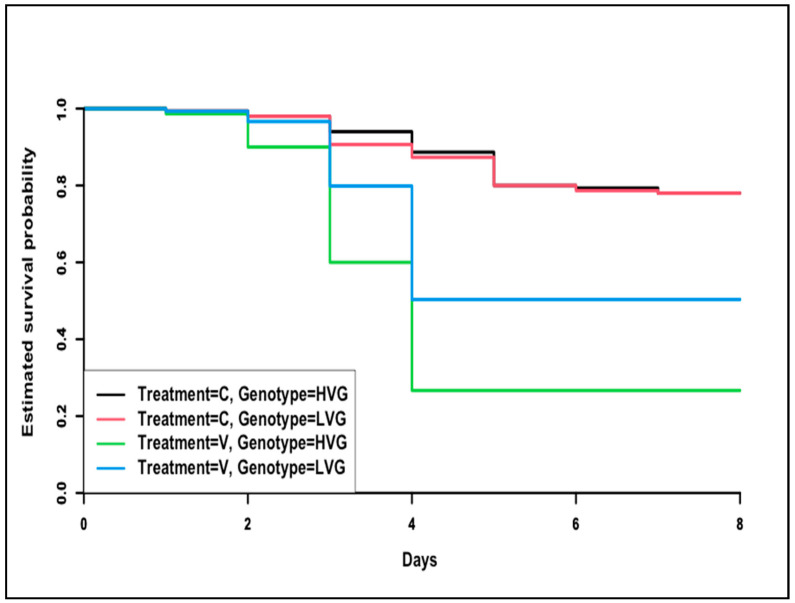
Survival probability of worker honey bees of the third generation of selection for high and low *Varroa* population growth (HVG and LVG, respectively) that were exposed (V) or not exposed (C) to *Varroa* in cages. The Kaplan–Meier method and the log-rank/Mantel-Cox post hoc test were used to determine which curves were significantly different from each other.

Survival analysis showed that there were no differences in the probability of survivorship of non-parasitized HVG and LVG bees (*p* > 0.05, Figure 5). However, *Varroa* parasitism significantly reduced the survivorship of both HVG and LVG bees compared to non-parasitized bees with significantly lower survivorship of parasitized HVG bees compared to LVG bees (χ^2^ = 137, df = 3, *p* < 0.01). By eight days, the survivorship of *Varroa*-parasitized bees was 46.0% lower in HVG than in LVG bees.

## 4. Discussion

In this study, honey bee colonies were selected for *Varroa* population growth rather than for *Varroa* infestation rates. Measuring infestation levels in adults and brood at the end of summer can be less accurate than growth rates, as mite levels are affected by many parameters, such as colony status, amount of brood, season, or climate [27]. Therefore, mite populations were measured at two time points as it has been shown that determining *Varroa* growth with at least a 10-week difference compensates for those factors by observing the dynamics of mite reproduction [32].

Selection for *Varroa* population growth in each generation of the LVG genotype had a more noticeable effect than selection in each generation of the HVG genotype. In addition to reducing the mean *Varroa* population growth with each generation, selection for LVG reduced the number of outliers and the skewing of variation for higher *Varroa* population growth than HVG colonies. Thus, part of the selection process appears to have been to make LVG colonies more uniform as well as reduce *Varroa* population growth. By the third generation, the selection of colonies for high or low *Varroa* growth resulted in LVG colonies in which mite populations grew almost 10-fold less than in HVG colonies. Infestation rates of both brood and adult bees in late summer were also significantly lower in LVG colonies by the third generation. It is not clear why the level of variation for the infestation of HVG colonies was similar to that of LVG values for brood, but not for adults. However, greater variability of *Varroa* resistance in brood may explain why only the first two generations of brood, and not subsequent generations, selected for low *Varroa* growth were more resistant than those selected for high *Varroa* growth [33]. It is also possible that between the second and third generations, mite levels had already reached a threshold in the LVG genotype that caused no significant damage to the colony, thus decreasing selection pressure for further reduced mite levels [34]. Additionally, it is possible that selection resulted in LVG and HVG bees responding to mite population growth differently due to environmental conditions. However, both genotypes were selected under the same environmental conditions, and the climate in southern Ontario is similar to that of Northern Europe where breeding programs for *Varroa* resistance have been conducted with no reports of climatic effects on selection for *Varroa* growth [35]. Overall, it seems that more consistent results can be achieved by selecting LVG than HVG bees, and even though the *Varroa* population growth of the third generation for the LVG genotype was not significantly lower than that of the second generation, the third generation of LVG bees benefited by having less variation from the mean (i.e., more consistency among LVG colonies).

Compared to the 89.7% reduction in mite growth for LVG compared to HVG colonies in this study, Lodesani et al. [36] reported 54.1% less *Varroa* growth with selection for low and high *Varroa* population growth over three generations. Emsen et al. [25] found a 91.2% mite population reduction selecting for low and high *Varroa* growth, but just in one generation. Using the same approach for ten generations selecting only for low *Varroa* growth, Harris et al. [32] achieved a *Varroa* population growth 35% lower than that of the initial colony populations by the third generation, 99% lower by the fifth generation but then 56% lower by the tenth generation. Selecting for only low *Varroa* growth for three generations, Rinderer et al. [21] reported that the mite population grew 84.6% lower than those of unselected colonies. Thus, the differences in *Varroa* population growth in this study were similar to or higher than in most of those studies, particularly when selecting for the same number of generations, although it is not clear that more generations of selection increase the level of resistance. The considerable range of reductions between the studies could be due to different environmental conditions during the selection process, which can affect *Varroa* growth [32]. Another possibility is that the genes selected for increased resistance in the starting genetic material were different, which is difficult to assess unless molecular markers could be used to compare if the same genomic regions were selected in each study.

DWV levels of LVG bees were significantly lower than those of bees from HVG bees in the third generation, reaching a 40% reduction. Similarly, previous studies have shown that LVG-selected bees have 47% less DWV compared with HVG bees [37], and lower levels of DWV in *Varroa*-resistant colonies [38], both of which support our findings. Higher DWV levels have been correlated with higher *Varroa* loads [9,39]. Possible mechanisms involved in viral level differences could include less feeding by the mite in LVG bees, thus reducing the amounts of DWV transmitted, and resistance to DWV multiplication, such as RNAi [40]. Further analysis of mechanisms behind reduced DWV levels, such as levels in caged parasitized bees, could help determine if antiviral immunity is involved. In addition, the assay used in this study is for DWV A [29], rather than DWV B or DWV C. It is possible that those variants of the virus were also present, which could be studied in the future.

Despite parasitized bees of the LVG genotype surviving significantly longer, the summer adult bee populations of the third generation of LVG and HVG colonies did not differ. None of the other studies that also selected colonies for LVG assessed colony populations, and thus it is not possible to compare to this study. However, colonies selected for *Varroa*-sensitive hygiene did not differ in bee populations compared to non-selected colonies [20], and colonies with natural resistance to *Varroa* did significantly differ in population compared to non-selected ones [41,42]. This is surprising as mites significantly shorten the lifespan of honey bees [1]. However, it is possible that the queen compensates for the loss of parasitized bees by increasing its egg-laying rate, thus maintaining the colony population. It has been reported that the egg-laying rate of queens can be adjusted based on colony size [43]. Nevertheless, this and other plausible hypotheses warrant further investigation because the reasons why LVG and HVG colonies do not differ in their populations are currently not well understood.

Winter mortality of LVG colonies was approximately three times lower than that of HVG colonies in the third generation. While no previous study of selection for LVG has examined winter colony mortality, studies selecting for either grooming or hygienic behaviors have shown an overwinter survival rate three times higher than that of non-selected colonies [18,20]. Also, Pol-line honey bee colonies that are considered *Varroa* resistant had an overwinter survival rate two times higher than that of a susceptible genotype [44]. Thus, selection for LVG decreases winter colony mortality, but the level may depend upon the degree of winter stress on the bees, which is affected by weather conditions, such as extreme cold periods in mid to late winter [44].

The survival of non-parasitized honey bees was not different between LVG and HVG bees. However, when bees were parasitized by *Varroa* for eight days, the survivorship of HVG bees was 46% lower than that of LVG bees. This indicates that LVG bees were more tolerant than HVG bees to *Varroa* parasitism, and LVG selection not only resulted in lower numbers of mites in the colonies but also in bees that could survive longer when parasitized. This is the first study to show that selection for LVG makes parasitized bees better able to tolerate the detrimental effects of *Varroa*. Since both the LVG and HVG bees had mites on them, bees of both genotypes experienced cuticle puncture, fat body and hemolymph loss, mite saliva protein injection, and other detrimental factors associated with *Varroa* parasitism [1]. LVG bees likely had some cellular and humoral responses limiting detrimental *Varroa* effects that were lower or missing in HVG bees. In addition to a possibly stronger immune response, LVG bees may be more stress-resistant than HVG bees, which warrants further investigation.

Future work could examine the mechanisms of *Varroa* resistance selected in this study. Lodesani et al. [36] postulated that selecting for LVG strongly correlated with the amount of brood reared, but not with hygienic behavior, grooming behavior, or mite reproduction. In contrast, Rinderer et al. [21] proposed that selection correlated with increased hygienic and grooming behaviors, while Seltzer et al. [45] suggested that breeding for hygienic behavior makes bees more resistant to *Varroa*. Others have proposed that mite resistance may be due to reduced *Varroa* reproduction [25,32,46]. This could be related to differences in humoral immunity, such as the *Varroa*-tolerant colonies having more differentially expressed genes than susceptible colonies, including those for olfactory signaling, detoxification, and exoskeleton formation [47]. Thus, it is desirable that more factors are examined during the selection process to better understand the range and type of mechanisms involved. It is likely that selecting the LVG trait has simultaneously included more than one resistance factor against the parasite.

## 5. Conclusions

A selective breeding program based on differential population growth of *Varroa destructor* resulted in two distinct honey bee genotypes that show differences in their response to the parasite. After three generations of selection, the LVG and HVG populations of honey bees significantly differed in *Varroa* infestation rates, population growth, DWV levels, and colony and individual survivorship. Thus, selecting honey bee populations for low *Varroa* population growth resulted in multiple beneficial effects. Because the resistance to *Varroa* seems to be a trait mainly transmitted by the queen [48] and because the methodology used to select LVG colonies is relatively simple, it could be easily and economically implemented by queen breeders [49]. Breeding for LVG appears to have great potential in improving honey bee management, providing an effective means of controlling *Varroa* with less reliance on the use of acaricides.

## Data Availability

The data from this study will be provided by the corresponding author upon reasonable request.
